# The complete mitochondrial genomes of *Ephemera serica* (Ephemeroptera: Ephemeridae) and phylogenetic analysis

**DOI:** 10.1080/23802359.2022.2044401

**Published:** 2022-03-09

**Authors:** Lili Wang, Bo Li, Jian Jiang, Xiaoli Tong

**Affiliations:** a Department of Entomology, College of Plant Protection, South China Agricultural University, Guangzhou, China; b Engineering Research Center of Biological Control, Ministry of Education, Guangzhou, China

**Keywords:** Mayfly, *Ephemera*, mitogenome, phylogeny

## Abstract

In the present research, the mitochondrial genome of *Ephemera serica* was sequenced through next generation sequencing methods and its phylogenetic position in Ephemeroptera was analyzed. Total mitochondrial genome is 15,004 bp in length, and contains 13 protein coding genes, two ribosomal RNA genes, and 22 transfer RNA genes. Mitogenomic phylogeny trees were constructed including 45 species from 13 families. The results show that *E. serica* is closely related to *E. rufomaculata*.

The burrowing mayfly genus *Ephemera* L. (Ephemeroptera: Ephemeridae) contains 68 species worldwide and is mainly distributed in Oriental Region (Hwang and Bae 2008). *Ephemera* species have important position in systematics and phylogeny of Ephemeroptera. However, so far, only four mitochondrial genomes of *Ephemera* are available to study the phylogenetic relationship of Ephemeroptera (Lee et al. 2009; Song et al. 2019; Yu et al. 2021). Sparse studies conducted on the genus *Ephemera* have blocked our understanding of the phylogenetic relationship of the genus with other mayfly groups. In this study, we provide a new mitogenome data of *Ephemera serica* Eaton, 1871, one of most encountered *Ephemera* species in southern China, for further analyzing the mayfly phylogeny.

Nymphs of *Ephemera serica* were collected from an inflow stream of the Longdong Reservoir (113.3976°E, 23.2336°N), Guangzhou, China. The voucher specimen of *Ephemera serica* (the voucher no. E3) was deposited in the Insect Collection, South China Agricultural University (SCAU), Guangzhou, China (Xiaoli Tong, xtong@scau.edu.cn). The genomic DNA of the specimen was extracted using phenol–chloroform method. Library prepration was done using TruSeq DNA sample Preparation kit (Vanzyme, China). DNA data were obtained by Illumina Hiseq 2500 (Illumina, USA) with a PE150 strategy (2 × 150 base paired-end reads) and deposited in GenBank (OK018134). Base composition was analyzed in MEGA 7.0 (Kumar et al. 2016).

*Nesomachilis australica* Tillyard, 1924 and *Pedetontus silvestrii* Mendes, 1993 were selected as outgroups. All 13 PCGs (protein coding genes) and two rRNA genes were aligned individually by MAFFT (Katoh and Standley 2013). Gblocks was used to detect the conserved regions with default settings (Talavera and Castresana 2007). The best-fit models were selected using PartitionFinder2 (Lanfear et al. 2017) by gene types based on Bayesian information criterion (BIC). Phylogenetic analyses were performed using MrBayes 3.2.6 (Ronquist et al. 2012) and IQ-TREE (Guindon et al. 2010; Minh et al. 2013; Nguyen et al. 2015). These analyses were all implemented in the PhyloSuite (Zhang et al. 2020).

The mitochondrial genome of *E. serica* was 15,004 bp in length, containing 13 PCGs and two ribosomal RNA genes and 22 transfer RNA genes, but the control region failed to be covered. The overall base contents are 36.2% A, 36.6% T, 16.4% C, and 10.8% G, indicating contents of AT 72.8% and GC 27.2%.

The phylogenetic tree in Figure 1 reveals the relationships of Ephemeroptera that was generated through Bayesian inference (BI) method, and additional maximum likelihood (ML) tree with the identical topology shows only the bootstrap values, except the relationship within Heptageniidae.

**Figure 1.**
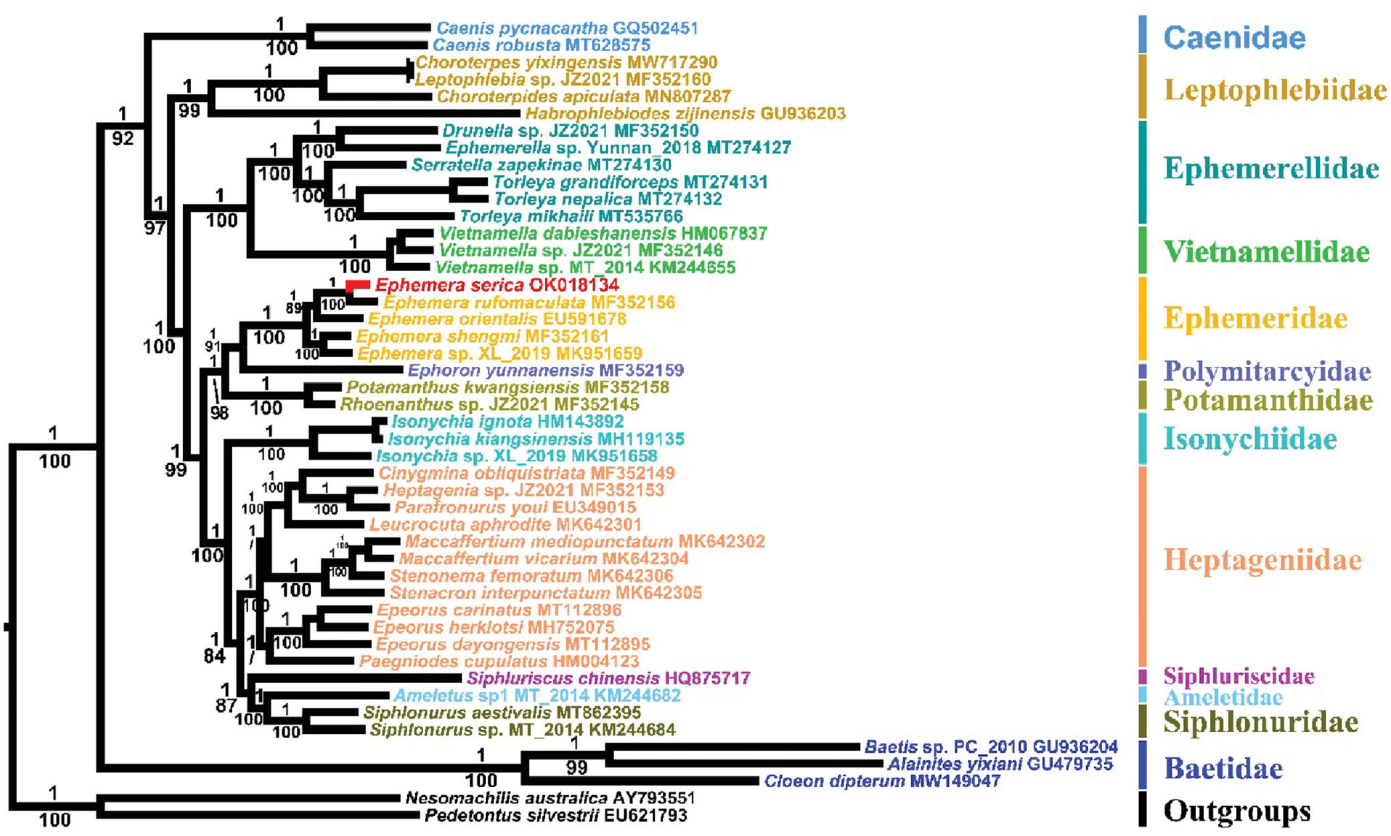
Phylogenetic tree of Ephemeroptera based on 13 PCGs and 2 rRNA genes, inferred using MrBayes (BI) and IQ-tree (ML). The values above and below the branches are the Bayesian posterior probability and maximum-likelihood ultrafast bootstrap values, respectively.

All nodes are strongly supported, except for few nodes in ML analyses with bootstrap values lower than 90. The results show that *E. serica* is closely related to *E. rufomaculata*, and three burrowing mayfly families, Ephemeridae, Polymitarcyidae, and Potamanthidae cluster together, which is consistent with the other previous works (Ogden et al. 2009; Yu et al. 2021). Due to unpublished paper, the data of *Siphlonurus immanis* (Siphlonuridae) is not included in the current study. Interestingly, *S.immanis* was clustered a sister clade with *Ephemera orientalis* (Ephemeridae) in the study of Guan et al. (2021). As a result, Siphlonuridae was divided into two branches, which suggested that Siphlonuridae was recovered as a polyphyly (Guan et al. 2021). However, in the present study, Siphlonuridae is supported as monophyletic, which forms a sister clade with Ameletidae. Besides, the unstable position of Isonychiidae still remains (Ogden et al. 2009; Guan et al. 2021). Resolving these issues require extensive taxon sampling of mitogenomes or larger dataset of related taxa.

## Data Availability

The data that support the findings of this study are openly available in GenBank of NCBI at (https://www.ncbi.nlm.nih.gov) under the accession number of OK018134. The associated BioProject, SRA, and Bio-Sample numbers are PRJNA764453, SRR16003088, and SAMN21502591 respectively.
